# Supplementary material for: Physical Activity and Sedentary Patterns of Pregnant Women in Southern Spain and the Relationship with Sociodemographic and Obstetric Characteristics: A Cross-Sectional Study
Source: Healthcare (Basel). 2025 Jun 13;13(12):1423. doi: 10.3390/healthcare13121423 (PMC12192721; doi:10.3390/healthcare13121423)
Supplement: Supplementary file 1 [file healthcare-13-01423-s001.zip › Table S1.pdf]

Table S1. Variability of physical and sedentary activities during pregnancy according to sociodemographic and somatometric characteristics.

Variables / Categories	Leisure PA min/week			Work PA min/week			Travel to and from places PA min/week			MET minutes/week			Sedentary minutes/day		
	Mean	SD	Sign.	Mean	SD	Sign.	Mean	SD	Sign.	Mean	SD	Sign.	Mean	SD	Sign.
Age			p=0.06			p=0.17			p=0.6			p=0.18			p=0.29
Less than 30 years	182.86	221.12		770.08	1,193.63		108.28	233.72		5,316.81	7,536.17		215.80	126.82	
30 to 35 years	176.68	197.66		528.91	934.35		86.62	215.12		3,677.20	5,254.22		267.86	193.74	
More than 35 years	259.61	319.71		509.61	965.19		97.13	190.35		4,262.84	5,762.11		225.39	161.33	
Educational level			**p<0.05**			**p<0.01**			p=0.28			**p<0.01**			**p<0.01**
No studies[a]	20.00	40.00	c, d	727.50	1,337.69		0.00	0.00		5,720.00	10,750.70		105.00	51.96	c, d
Compulsory schooling [b]	193.19	255.03	d	916.42	1,233.32	c, d	100.56	239.02		6,294.78	7,813.60	c, d	195.90	127.05	c, d
Pre-university studies[c]	215.06	198.56	a	330.70	758.30	b	92.53	178.85		2,707.59	3,448.23	b	254.14	166.90	a, b
Higher education [d]	243.95	310.54	a, b	318.49	720.22	b	100.17	193.53		2,892.44	4,014.44	b	280.97	192.05	a, b
Currently studying			**p<0.05**			**p<0.01**			p=0.12			p=0.73			p=0.19
No	198.06	235.31		658.06	1,085.14		95.99	215.94		4,687.38	6,570.30		230.67	162.83	
Yes	292.46	375.01		244.21	602.12		106.67	183.74		2,897.54	3,673.28		257.11	163.52	
Employment situation			p=0.87			**p<0.05**			p=0.27			p=0.12			p=0.38
Full time [e]	212.44	240.42		592.18	1,068.96	g	76.28	192.99		4,132.05	5,797.18		268.74	201.44	
Part time [f]	228.66	331.38		654.51	1,010.48	g	102.20	204.62		4,869.27	6,872.31		209.09	129.92	
Unemployed [g]	195.66	211.93		354.55	937.02	e, f, h	130.41	248.83		3,180.18	5,285.89		225.00	127.12	
Other [h]	203.93	263.30		777.87	1,075.26	g	106.13	218.88		5,767.73	7,150.25		194.07	115.87	
Size of place of residence			p=0.51			**p<0.05**			p=0.66			**p<0.05**			p=0.13
Up to 5,000 inhabitants [i]	225.11	227.89		688.89	1,178.00		139.00	351.39		5,446,67	8,075.07		196.33	129.03	
5,001 to 20,000 inhabitants [j]	256.71	352.68		764.63	998.78	l	93.35	166.48		5,278.29	5,911.79	k, l	199.76	125.89	
20,001 to 50,000 inhabitants [k]	191.25	227.36		583.90	1,000.03		73.42	156.57		4,247.50	5,947.52	i	246.21	169.26	
More than 50,000 inhabitants [l]	200.37	237.57		459.50	1,042.73	i	112.17	222.22		3,655.80	6,004.34	i	259.63	183.45	
In a relationship			p=0.92			p=0.75			p=0.21			p=0.8			p=0.37
Yes	212.44	263.09		593.27	1,033.72		98.34	211.93		4,389.59	6,197.47		234.41	163.48	
No	165.00	158.03		980.00	1,697.41		0.00	0.00		8,500.00	13,010.50		260.00	91.65	
Current language spoken at home			p=0.78			p=0.85			p=0.56			p=0.87			p=0.53
Spanish	211.03	263.36		599.30	1,040.26		99.78	218.91		4,460.32	6,319.26		236.10	164.38	
Other (Including bilinguals)	221.58	256.06		569.21	1,025.60		77.50	121.57		4,070.00	5,699.70		221.05	151.12	
Language spoken at home as a child			p=0.45			**p<0.05**			p=0.2			p=0.07			**p<0.05**
Spanish	211.32	266.63		564.62	1,025.29		102.82	219.05		4,314.52	6,283.85		239.28	164.97	
Other (including bilinguals)	219.31	219.62		900.83	1,118.29		46.81	99.13		5,457.78	5,951.16		189.58	136.31	
Country of birth			p=0.84			**p<0.05**			p=0.74			p=0.08			p=0.38
Spain	213.78	268.38		568.80	1,027.84		99.88	216.43		4,309.52	6,243.45		235.83	162.32	
Another country	193.28	185.23		897.19	1,110.96		72.19	142.81		5,655.63	6,346.92		221.25	172.21	
Body Mass Index (BMI)			p=0.29			p=0.75			p=0.08			p=0.67			p=0.15
Underweight	192.00	245.19		546.00	1,220.89		462.00	558.68		6,984.00	10,340.35		222.00	162.39	
Normal weight	237.91	290.53		536.35	976.92		85.21	187.09		3,923.91	5,552.11		240.82	165.78	
Overweight	188.06	234.35		598.17	1,036.30		99.61	237.23		4,607.93	6,479.77		245.78	165.90	
Obesity	184.10	233.09		743.46	1,176.17		99.49	173.46		5,182.05	7,209.59		208.46	153.80	

Note: Mann Whitney U test was used to assess releationhip with physical activity and sedentary variables..
