# Supplementary material for: Physical Activity and Sedentary Patterns of Pregnant Women in Southern Spain and the Relationship with Sociodemographic and Obstetric Characteristics: A Cross-Sectional Study
Source: Healthcare (Basel). 2025 Jun 13;13(12):1423. doi: 10.3390/healthcare13121423 (PMC12192721; doi:10.3390/healthcare13121423)
Supplement: Supplementary file 1 [file healthcare-13-01423-s001.zip › Table S2.pdf]

**Table S2.** Variability of physical and sedentary behaviour during pregnancy according to obstetric characteristics

| Variables / Categories | Leisure PA min/week | | | Work PA min/week | | | Travel to and from places PA min/week | | | MET – min/week | | | Sedentary min./day | | |
|---|---|---|---|---|---|---|---|---|---|---|---|---|---|---|---|
| | Mean | SD | Sign. | Mean | SD | Sign. | Mean | SD | Sign. | Mean | SD | Sign. | Mean | SD | Sign. |
| Gravidity (number of pregnancies including the current one) | | | **p<0.01** | | | p=0.07 | | | p=0.64 | | | p=0.64 | | | **p<0.05** |
| Primigravida | 254.33 | 242.74 | | 470.85 | 939.63 | | 98.23 | 222.17 | | 3,863.18 | 5,482.26 | | 251.91 | 160.35 | |
| Multigravida | 187.27 | 270.61 | | 670.54 | 1,086.39 | | 97.19 | 205.09 | | 4,749.42 | 6,655.75 | | 224.52 | 163.99 | |
| Health problems during pregnancies ended in live birth | | | p=0.13 | | | p=0.9 | | | p=0.97 | | | p=0.43 | | | p=0.96 |
| No | 199.37 | 300.35 | | 621.20 | 1,035.87 | | 103.36 | 227.58 | | 4,647.42 | 6,571.48 | | 227.83 | 170.32 | |
| Yes | 123.17 | 198.20 | | 580.34 | 997.64 | | 96.33 | 197.37 | | 3,506.00 | 5,120.43 | | 205.50 | 129.04 | |
| Pregnancy planning | | | p=0.34 | | | p=0.28 | | | p=0.51 | | | p=0.51 | | | p=0.6 |
| No | 195.67 | 243.24 | | 646.52 | 1,002.53 | | 121.92 | 274.21 | | 4,806.36 | 6,271.93 | | 213.03 | 125.34 | |
| Yes | 217.83 | 268.26 | | 580.58 | 1,050.89 | | 90.43 | 188.06 | | 4,308.81 | 6,265.72 | | 241.76 | 172.40 | |
| Assisted reproduction pregnancy | | | p=0.44 | | | p=0.12 | | | p=0.77 | | | p=0.25 | | | p=0.15 |
| No | 216.10 | 271.69 | | 623.72 | 1,098.43 | | 88.25 | 186.58 | | 4,580.71 | 6,615.44 | | 238.72 | 173.25 | |
| Yes | 243.19 | 251.74 | | 267.50 | 571.08 | | 66.67 | 134.27 | | 2,309.44 | 2,367.26 | | 270.83 | 170.84 | |
| High-risk pregnancy consultation | | | p=0.36 | | | p=0.68 | | | p=0.37 | | | p=0.9 | | | p=0.42 |
| No | 217.13 | 268.96 | | 585.37 | 1,043.02 | | 98.13 | 208.48 | | 4,378.80 | 6,292.21 | | 231.08 | 159.00 | |
| Yes | 184.75 | 223.20 | | 655.00 | 1,014.05 | | 94.58 | 227.66 | | 4,653.33 | 6,095.47 | | 253.58 | 183.25 | |
| Trimester of pregnancy awareness | | | p=0.47 | | | **p<0.01** | | | p=0.94 | | | **p<0.01** | | | p=0.48 |
| First trimester | 212.09 | 262.55 | | 569.92 | 1,020.82 | | 97.49 | 211.10 | | 4,273.58 | 6,154.53 | | 235.24 | 163.21 | |
| Second trimester | 162.00 | 272.98 | | 2,124.00 | 741.37 | | 123.00 | 266.73 | | 13,836.00 | 6,577.54 | | 198.00 | 174.41 | |
| Professional health care during pregnancy | | | p=0.73 | | | p=0.62 | | | p=0.39 | | | p=0.93 | | | p=0.96 |
| No | 315.00 | 445.48 | | 940.00 | 1,329.36 | | 420.00 | 593.97 | | 10,300.00 | 14,566.40 | | 210.00 | 127.28 | |
| Yes | 211.53 | 261.92 | | 594.50 | 1,037.71 | | 95.88 | 208.33 | | 4,390.92 | 6,212.02 | | 234.74 | 163.28 | |

Note: Mann Whitney U test was used to assess releationhip with physical activity and sedentary variables.
